# Two Is Better Than One: Evidence for T-Cell Cross-Protection Between Dengue and Zika and Implications on Vaccine Design

**DOI:** 10.3389/fimmu.2020.00517

**Published:** 2020-03-25

**Authors:** Krishanthi S. Subramaniam, Suzannah Lant, Lynsey Goodwin, Alba Grifoni, Daniela Weiskopf, Lance Turtle

**Affiliations:** ^1^NIHR Health Protection Research Unit in Emerging and Zoonotic Infections, Centre for Global Vaccine Research, Institute of Infection and Global Health, University of Liverpool, Liverpool, United Kingdom; ^2^Division of Vaccine Discovery, La Jolla Institute for Immunology, La Jolla, CA, United States; ^3^Tropical and Infectious Disease Unit, Liverpool University Hospitals, Liverpool, United Kingdom

**Keywords:** dengue, Zika, T-cells, cross-reactivity, vaccine, epitope, animal models

## Abstract

Dengue virus (DENV, family *Flaviviridae*, genus *Flavivirus*) exists as four distinct serotypes. Generally, immunity after infection with one serotype is protective and lifelong, though exceptions have been described. However, secondary infection with a different serotype can result in more severe disease for a minority of patients. Host responses to the first DENV infection involve the development of both cross-reactive antibody and T cell responses, which, depending upon their precise balance, may mediate protection or enhance disease upon secondary infection with a different serotype. Abundant evidence now exists that responses elicited by DENV infection can cross-react with other members of the genus Flavivirus, particularly Zika virus (ZIKV). Cohort studies have shown that prior DENV immunity is associated with protection against Zika. Cross-reactive antibody responses may enhance infection with flaviviruses, which likely accounts for the cases of severe disease seen during secondary DENV infections. Data for T cell responses are contradictory, and even though cross-reactive T cell responses exist, their clinical significance is uncertain. Recent mouse experiments, however, show that cross-reactive T cells are capable of mediating protection against ZIKV. In this review, we summarize and discuss the evidence that T cell responses may, at least in part, explain the cross-protection seen against ZIKV from DENV infection, and that T cell antigens should therefore be included in putative Zika vaccines.

## Introduction

**Figure 1:**
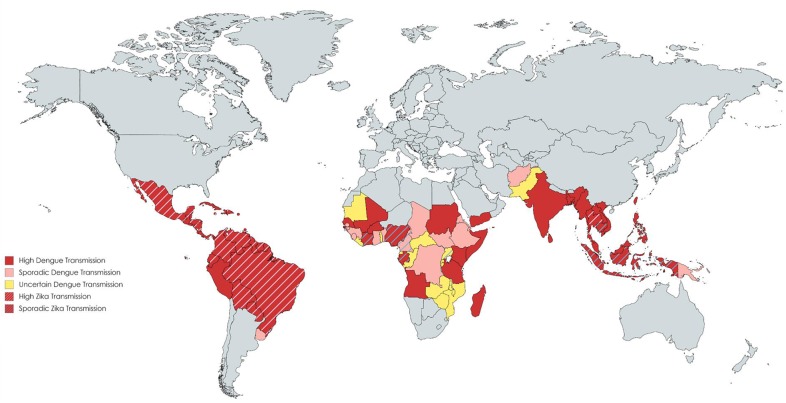
Distribution of countries with Dengue and Zika transmission. Data compiled using the CDC yellow book and HealthMap©. Each color reflects a varying transmission rate from high dengue transmission (in red) to uncertain dengue transmission (in yellow). Countries with cross-hatched lines reflect those with high or sporadic Zika transmission.

During the last two decades, the rate of infections with flaviviruses, particularly dengue virus (DENV) and Zika virus (ZIKV), has risen significantly (Figure 1). At present, half of the world's population is considered at risk for DENV and cases of ZIKV continue to be reported globally, including the first local cases in southern Europe (1, 2). DENV and ZIKV are spread via the bite of infected mosquitoes, *Aedes spp*., whose expanding ecological niches beyond the tropical and sub-tropical regions pose a major public health threat (3). Infection with DENV can present with a spectrum of clinical manifestations ranging from an asymptomatic illness to an acute fever/arthralgia/rash (dengue fever, DF) that usually is self-limiting, to more severe disease (dengue hemorrhagic fever/dengue shock syndrome, DHF/DSS) that is characterized by vascular leakage and/or hemorrhage (4). ZIKV causes a similar febrile illness that is often mild, with the exception of rare cases of neurological disease, such as Guillain-Barré syndrome (5). In pregnancy, ZIKV infection is associated with adverse fetal/neonatal outcomes such as congenital Zika syndrome (CZS) (6). Given that these viruses share similar geographic distributions and high sequence homology, immunological cross-reactivity between DENV and ZIKV is a well-recognized and unsurprising phenomenon. In this review, we will focus on the role of T cells during DENV and ZIKV infections in humans and in animal models, summarizing the major findings, discussing how cross-reactivity might impact immunity, and providing evidence why incorporating T cell epitopes into vaccine design is favorable.

## Virology

There are four dengue viruses, DENV1-4, which are antigenically distinct (hence called serotypes) and possibly represent four distinct introductions into humans from the sylvatic cycle in non-human primates (7). On occasion several serotypes can circulate concomitantly within endemic areas, or as individual serotypes in sequence (8). A primary dengue infection generally results in lifelong immunity against the same serotype, although homotypic DENV re-infections have also been described (9). DENV infections can generate cross-reactive, poorly neutralizing antibodies that bind the other serotypes (10). Upon secondary infection with a heterologous DENV serotype, there is then a risk of severe disease, thought to be mediated via a mechanism called antibody-mediated enhancement (ADE) (11, 12). ADE arises when antibodies against one serotype can bind to, but not fully neutralize, another DENV serotype. These virus-antibody complexes can bind to the Fcγ receptors on the surface of mononuclear phagocytes enhancing viral entry and facilitating viral replication (13).

ZIKV has three genotypes, East African, West African and Asian (14). Recent Zika outbreaks have indicated a role for pre-existing immunity against DENV to modulate ZIKV infection (15). Data from a large pediatric cohort in Nicaragua found that prior DENV infection reduced the risk of symptomatic ZIKV infection by about one third (16) and in a Brazilian cohort high pre-existing antibody titers to DENV were associated with reduced risk of ZIKV infection and symptoms (17). Furthermore, protection against congenital Zika syndrome was shown to be associated with prior DENV immunity (18).

## Viral Structure

The flavivirus virion is enveloped, and contains a single-stranded, positive-sense RNA that is ~11 kb size. The viral genome encodes three structural proteins [capsid, precursor membrane (prM) and envelope (E)] involved in virion assembly and seven non-structural (NS) proteins (NS1, NS2a, NS2b, NS3, NS4a, NS4b, and NS5) that function in the viral life cycle (19). The canonical role of NS proteins is in viral replication where with host factors they function in the assembly of the membrane-bound multi-protein replication complex (RC). NS proteins are also the target of most of the flavivirus CD8 T cell epitopes (20–22). A mature flavivirus particle has a well-organized outer glycoprotein shell with an icosahedral T = 3 symmetry, a host derived lipid bilayer membrane and a poorly defined inner nucelocapsid core (23). Flavivirus particles can assume various morphologies (immature, mosaic-like, and mature) that vary between flaviviruses and have important implications on antibody binding specifically regarding the availability and accessibility of epitopes (24) [reviewed in (25)].

## Antibody-Mediated Immunity

Neutralizing antibody plays a crucial role in immunity to flaviviruses. Animal models show that robustly neutralizing monoclonal antibodies are sufficient for protection against many flaviviruses (24, 26, 27). However, antibody responses against flaviviruses can also be notoriously cross-reactive and the neutralization potential of these antibodies can vary considerably. The neutralizing antibody response is directed against the E protein, but responses against other proteins such as prM and NS1 also form a significant fraction of the response after both DENV and ZIKV infections (25, 28–30).

In recent years, much has been learned about anti-flavivirus antibody responses by cloning antibodies from infected or previously infected humans. These studies have demonstrated that, while many different classes of antibody are made, those which most potently neutralize frequently recognize quaternary epitopes on the viral surface and bind across multiple Envelope proteins (24, 31–33). The probable mechanism of neutralization by antibodies against quaternary epitopes is through interfering with viral fusion by locking the particle in a non-fusogenic form (24). Although some of these antibody classes can neutralize all four DENV serotypes and ZIKV (34, 35), they may not be durable in humans and their effectiveness may wane with time (36). In fact, a period of cross-protection exists even after a primary DENV infection, as observed by Sabin who found that subjects re-challenged with heterotypic DENV infection were protected if the re-challenge occurred 2–3 months after the original infection (37).

A major epitope recognized by the human antibody response is the viral fusion-loop of E domain II (25). Fusion loop antibodies are highly cross-reactive and strongly binding, but weakly neutralizing, not able to cross-neutralize other viruses, and can mediate ADE (38). During secondary flavivirus infections, the resulting antibody response can be predominantly focused upon the earlier infecting virus, a phenomenon known as “original antigenic sin (OAS);” and thus may be poorly neutralizing against the current infection (39). It may be challenging for new vaccines (such as those against ZIKV), when introduced in areas of intense flavivirus transmission, to protect if the balance of enhancing and neutralizing antibody is not optimal, or the development of new antibody responses is impaired (38). In addition, the development of congenital Zika syndrome has been linked to ADE (40) and could be due to the presence of cross-reactive fusion loop antibodies. Prior DENV immunity can protect against ZIKV, and, in the cases where inefficient antibody responses arise, possibly due to OAS, it might be that a cross-reactive CD8+ T cell response contributes to protection.

## T Cell Responses to DENV in Humans

**Figure 2:**
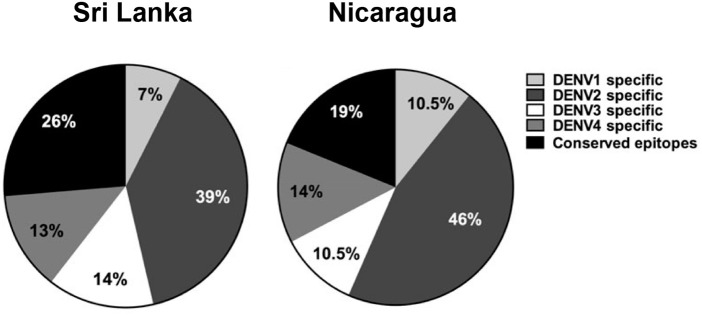
Distribution of CD8+ T cell epitopes across the four DENV serotypes in two clinically characterized cohorts from Sri Lanka and Nicaragua. Adapted from results in Gordon et al. (16), Weiskopf et al. (55).

**Figure 3:**
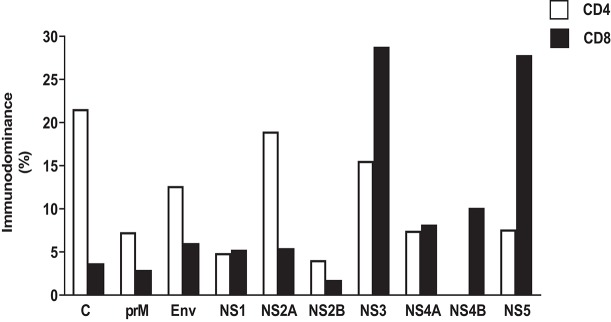
Immunodominant protein pattern of DENV-specific CD4 and CD8 T cell response based on IEDB data. HLA class I and class II restricted epitopes for CD4 and CD8 DENV-specific T cell responses, respectively, have been derived querying IEDB database (www.IEDB.org) on 02-Aug-2019. Epitope list was filtered using Immunobrowser, selecting epitope lists tested in at least 10 donors. Protein immunodominance was calculated as percentage of epitopes per protein per HLA restriction.

**Figure 4:**
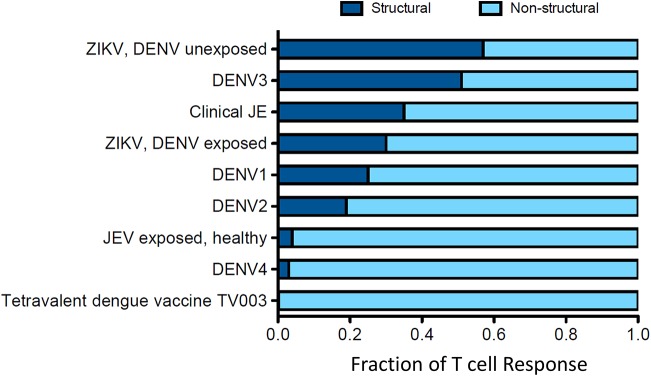
Distribution of the T cell response to structural and non-structural components is influenced by prior clinical history. Data for this figure was compiled from the following studies: Turtle et al. (20), Weiskopf et al. (21, 56), Grifoni et al. (54), and Delgado et al. (57). T cell responses are from subjects with natural infections or vaccine recipients. Responses were identified by ELISPOT and intracellular cytokine staining (ICS) assays.

Early work on cellular immunity to DENV demonstrated that T cell responses were readily detectable, and serotype cross-reactive responses of both CD4+ and CD8+ T cells were described (20–22, 41–46). The existence of serotype cross-reactivity at the level of individual T cell epitopes was found in both subjects given an experimental DENV vaccine (47, 48) and after natural exposure (49). In fact, a single DENV infection can elicit a cross-reactive T cell response against several serotypes (50), and the same T cell receptor (TCR) can recognize epitopes from multiple serotypes (51–53). Although variant epitopes may be recognized by the same TCR, the degree of overall serotype cross-reactivity is also likely to be influenced by the targeting of immunodominant responses, for example non-structural (NS) proteins are more highly conserved than structural proteins (Table 1). Responses biased toward sequences that are conserved between serotypes (possibly in NS proteins) give rise to higher potential for serotype cross-reactivity (55). The factors that determine whether responses are focused on conserved or variant epitopes are not known. However, interestingly, the pattern of conserved/serotype specific epitope recognition was remarkably similar in two different populations studied (Figure 2), implying that the factors underlying the phenomenon are not constrained to specific populations (55). The immunodominant targets of the T-cell response can vary between CD4+ and CD8+ T cells (Figure 3), and also between DENV serotypes (22, 55), as well as with exposure to other flaviviruses. Interestingly, the stimulus for the most cross-reactive T cell responses of all appears to be the tetravalent live attenuated DENV vaccine TV003, which includes the NS protein from DENV serotypes 1, 3, and 4, where the vast majority of the response is directed against NS proteins (Figure 4) (56). CD8+ T cells from TV003 vaccines can also cross-recognize ZIKV peptides, suggesting that the tetravalent DENV vaccination can induce T cell cross-reactivity across DENV serotypes and the closely related ZIKV (54).

**Table 1:** Percent homology across structural and non-structural proteins between Zika and DENV serotypes 1–4.

**ZIKA**											
	**Polyprotein%**	**C%**	**prM%**	**E%**	**NS1%**	**NS2A%**	**NS2B%**	**NS3%**	**NS4A%**	**NS4B%**	**NS5%**
DENV1	55	50	43	57	54	46	35	66	43	51	67
DENV2	56	41	41	55	54	44	41	67	52	53	67
DENV3	57	50	42	58	55	46	38	67	39	52	67
DENV4	57	49	47	56	54	45	41	67	44	49	68

*The results of this table was generated from data available in Grifoni et al. (54)*.

Demonstrating a clear role for DENV specific T cell responses in protection or disease has been more challenging. Initially, it was thought that the T cell response against DENV was pathological to the host. Some variant DENV epitopes may function as inefficient TCR agonists (58), and during acute disease CD8+ T cell responses can be more focused on variant epitopes from a previous infection (original antigenic sin) (51), possibly leading to less efficient responses. Moreover, many of these DENV specific T cells were found to be apoptotic (51). Some studies have demonstrated that *ex-vivo* T cell cytokine responses are greater with more severe disease, when tested shortly after disease onset (59, 60), suggesting a role for T cells in mediating excessive inflammation. In addition, degranulation of T cells [a surrogate marker for cytotoxicity (61)] was not greater in DHF (60, 62), implying that it is in fact the balance between cytokine production (pathological?) and killing (protective?) that may influence disease phenotype.

However, not all studies have shown a relationship between acute responses and disease phenotype, with some authors pointing out that the appearance of DENV specific T cells occurs after resolution of clinical outcomes in severe dengue (63). Furthermore, not all studies show a relationship between higher cytokine production and disease severity in DHF (64). In fact, when sampled early in the disease course, or as in one prospective study before disease onset, T cell cytokine production correlated with lower viremia and less severe disease (65, 66), implying a protective role for T cells. One study of HLA association with disease severity of dengue showed certain alleles to be protective whilst others were detrimental, potentially explaining why studies of T cells and disease severity give discrepant results (21, 67, 68).

## DENV/ZIKV T Cell Cross-Reactivity

**Figure 5:**
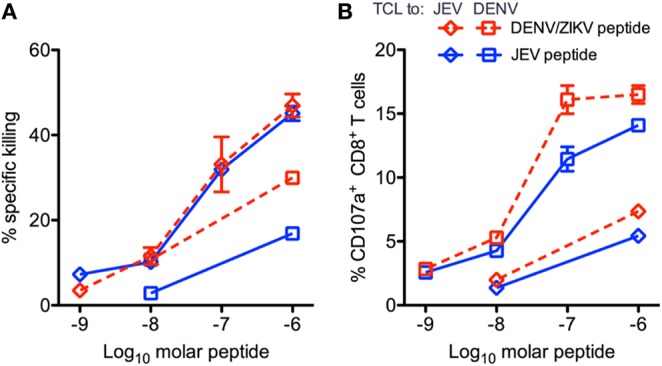
Peptide-pulsed, CFSE-labeled, HLA-matched targets were incubated with CD8+ T cell line effector cells, and the percent specific killing was measured by flow cytometry in response to JEV and DENV/ZIKV peptides. Percent killing **(A)** and the percent of CD107+ CD8+ T cells **(B)** are shown. Diamonds indicate T cell lines expanded with JEV peptide, and squares indicate lines expanded with DENV/ZIKV peptide. Assays were performed in duplicate for each T cell line/peptide pair. Error bars represent standard error of the mean. This figure was adapted from Turtle et al. (20).

Given the potential for T cell responses to DENV to be protective, at least in some circumstances, it is therefore possible that T cells primed by DENV could recognize ZIKV and be protective. Grifoni et al. found that in DENV exposed individuals, the T cell response to ZIKV is earlier, larger and exhibits greater cytotoxic capacity (54). In the same study it was shown that in two large cohorts from Sri Lanka and Nicaragua (54), the imprint of previous DENV exposure is clearly detectable, and that the resulting T cell response to Asian ZIKV was biased toward the non-structural proteins (Figure 4). Similarly, evidence of pre-existing flavivirus immunity has been shown to result in enhanced T cell responses directed to NS3 of DENV and African ZIKV (69). Whether such responses are protective is unknown, but two studies have demonstrated that short-term T cell cultures of flavivirus specific T cells are capable of killing targets pulsed with peptides that are found in ZIKV, indicating that they likely have anti-viral function (Figure 5) (20, 70). With DENV infection appearing to confer partial protection against Zika illness (16, 18), cross-reactive T cell responses may be one such mechanism by which this protection is mediated. Additionally, transcriptomic profiles of ZIKV-specific CD8+ T cells in DENV naïve or pre-exposed patients showed no qualitative differences in ZIKV- specific CD8+ T cell responses supporting the fact that cross-reactive T cell responses share the same protective phenotype observed after single flavivirus exposure (71). This phenomenon may also not be confined to ZIKV. There is some evidence that partial protection against Japanese Encephalitis (JE) appears to be conferred by prior DENV infection, with the number of JE cases lower than expected in areas with DENV outbreaks (72). Also it has been shown that the severity of JE is reduced by previous flavivirus infection (73).

These effects may be mediated by T cell responses (20). The degree to which T cell responses are targeted to structural vs. non-structural flavivirus proteins may vary according to previous exposure. For example, in the case of ZIKV, being affected by prior DENV infection biases the response toward the non-structural proteins (Figure 4). Responses against non-structural proteins tend to be more cross-reactive, meaning that previous dengue infection has the potential to bias the T cell response to ZIKV toward more cross-reactive epitopes. In an environment where multiple flavivirus exposure occurs, such epitopes, are likely to receive a great number of re-stimulations and may rise to the top of a “hierarchy of immunodominance.” Including such epitopes in vaccines, therefore, has merit in that such a vaccine may be made more effective if there is a degree of pre-existing immunity in the population, lowering the threshold for vaccine responses to be generated. Although further studies are required to unequivocally show that DENV-primed T cell responses can mediate protection against ZIKV in humans, mouse studies provide convincing evidence that T cells can mediate cross-protection.

## T Cell Responses in Mice and Non-Human Primates (NHP)

Mice are not natural hosts for flaviviruses as the murine type I IFN system provides a very effective defense, which thwarts viral dissemination and thus prevents them being useful models of severe disease phenotypes (74). As such, to establish a model of productive viral infection, which can be used to examine T cell function and test potential vaccine candidates, multiple strains of immunocompromised mice have been generated (74). These strains include mice deficient in either type I or type II or both IFN receptors, mice with STAT2 knocked-out, mice with mouse STAT2 replaced by human STAT2, and more nuanced models where type I IFN receptors are absent in specific cells or tissues (74). In combination with either mouse-adapted or human viral strains, this has established an infection model that closely, but not perfectly, mimics human disease. For DENV and ZIKV, strains that are commonly used either lack type I IFN receptor (IFNAR^−/−^ and A129) or both type I and II receptors (AG129 mice) (74). Human Leucocyte Antigen (HLA)-transgenic mice have also been used to model CD8+ and/or CD4+ T cell responses in flavivirus infection. Work in HLA-transgenic mice show a broad epitope repertoire, whilst some HLA variants, such as HLA-B^*^0702, have been specifically studied due to a known association with high T cell response frequency and magnitude in humans, as well as decreased susceptibility to severe dengue disease (75, 76).

Control of primary DENV infection in mice requires CD8+ T cells to a greater extent than CD4+ cells. In IFNAR^−/−^ mice, depletion of CD8+ cells was directly associated with increased viral burden in tissues that was not ameliorated by the transfer of serum or B cells (77). Protection in the study was mediated by increased cytotoxic activity in DENV-specific CD8+ T cells; activity which was further enhanced when mice received a peptide vaccination (77). However, challenge experiments in other mouse models (HEPG2-grafted SCID) find that DENV-specific CD8+ T cell responses were associated with reduced mortality, which suggests that T cells may contribute to disease severity in some instances, and prevent mortality in others (78). Crossing IFNAR^−/−^ mice with HLA transgenic mice showed that protective CD8+ T cell responses tended to be polyfunctional and principally targeted non-structural proteins such as NS3 and NS5, similar to that in humans (75). Responses targeted against NS proteins have also shown their protective potential in homotypic secondary DENV infections where wildtype mice primed with a non-lethal DENV2 strain ACS46 were challenged with a lethal encephalitic homotypic strain JHA1 (79). In this model, protective immunity was reduced when both CD4+ and CD8+ T cells were depleted. In most challenge models CD4+ T cells play an accessory and non-essential role in which they contribute to viral clearance when induced by immunization (80).

Models of heterologous DENV infection also demonstrate the importance of T cells during the anti-DENV response. Collectively these studies show that CD8+ responses can protect against heterologous DENV challenge in non-lethal (81) and lethal models (82). CD8+ T cell responses contribute to protection during heterotypic reinfection, whereas homotypic reinfection can be contained by neutralizing antibodies against the infecting serotype (81), as is believed to be the case in humans. Comparison of specific and cross-reactive T cell responses in IFNAR^−/−^ mice reveal that, despite their relatively low magnitude and avidity, cross-reactive CD8+ T cells from prior DENV exposure reduce viral load and exhibit a polyfunctional response in a manner comparable to that of serotype-specific cells (46). However, in a model of secondary DENV infection resulting in severe disease, cross-reactive CD4+ and CD8+ T cells were found to be pathogenic in wildtype mice infected with a non-mouse adapted DENV strain (83). In summary, the contribution of T cells to disease and protection in dengue mouse models is still not fully understood. The variability in current data are likely shaped by factors such as differences in mouse immune function, infection methods, strain difference, and experimental end-points.

Similar to observations for DENV, experiments in mice in which T cell responses are lacking, or on the other hand are enhanced (e.g., through peptide immunization or adoptive cell transfer), demonstrate a protective role for CD8+ T cells against ZIKV (84, 85). CD8+ T cell responses in primary ZIKV infection appear to be essential for immunity. In LysMCre^+^ IFNAR^fl/fl^ (type I IFN receptor absent only in myeloid cells) and IFNAR^−/−^ mice, ZIKV-immune CD8+ T cells protect against infection through cytotoxic, polyfunctional cellular responses (46, 86). However, in some instances, the resulting cytotoxicity may damage the host, in a tissue specific manner. For example, in IFNAR^−/−^ mice ZIKV infection of astrocytes results in a breakdown of the blood-brain barrier, allowing an influx of CD8+ T cells into the central nervous system (CNS) where they mediate apoptosis of ZIKV-infected neurons, but also results in severe neuropathology (87). Similarly, CD8+ cellular infiltration was also found in the CNS following ZIKV infection in C57/BL6 neonatal mice who developed hind limb collapse, cerebellar degeneration (88) and in the case of adult wildtype C57BL/6 mice, encephalitis (89). Whilst the CD8+ T cell response may be detrimental in the CNS, in IFNAR^−/−^ pregnant mice cross-reactive DENV-specific CD8+ cells are protective against ZIKV infection of the fetus, including the fetal central nervous system, and are associated with increased fetal growth and viability (90). The CNS may represent a special case, where infection in the absence of CD8+ T cells results in severe viral pathology, and in the presence of CD8+ T cells in immunopathology, with little difference in survival in either case, as is seen in Japanese encephalitis (91).

**Figure 6:**
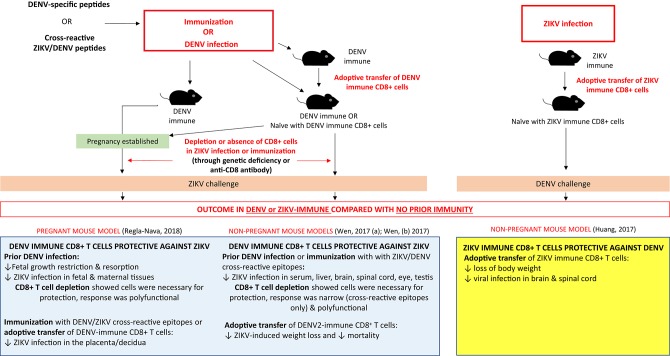
The CD8+ T cell response and impact of prior flavivirus immunity in secondary flavivirus infection in mice. Summary of experiments (infections, immunizations, adoptive cell transfer, and depletions) demonstrating the protective effect of prior DENV immunity on ZIKV challenge and prior ZIKV immunity on DENV challenge in pregnant and non-pregnant mouse models.

Responses to sequential DENV-ZIKV infection (summarized in Figure 6) share similarities with secondary heterotypic DENV infection. Firstly, DENV-immune CD8+ T cell responses—either from prior exposure, peptide immunization or transfer of DENV-immune CD8+ T cells—can protect against ZIKV infection (90, 92, 93). This is an important result, which corroborates human studies that demonstrate prior DENV immunity can reduce the risk of Zika infection (16). Likewise, prior exposure to DENV provided IFNAR^−/−^ mice protection against maternal and fetal ZIKV infection as compared with non-immune controls (90). As in heterotypic DENV, in mice as well as humans—the immunodominance pattern of the CD8+ T cell response to ZIKV infection was altered by prior DENV immunity and focused on conserved cross-reactive epitopes (93). ZIKV/DENV cross-reactive T cells performed comparably to ZIKV-specific T cells in viral load reduction in the serum and brain of knockout mice, which have key implications for ZIKV vaccine development.

Contrary to mice, non-human primates (NHP) are natural hosts for DENV and ZIKV. Several different primate models have been employed, including rhesus and cynomolgus macaques. In line with epidemiological observations in humans, experiments in NHP demonstrate that dengue immunity may curb Zika replication and potentially symptoms (94). Performing the order of viral challenge the other way around gave a similar result, where macaques with prior ZIKV immunity mounted strong humoral and cellular responses against DENV (95). Furthermore, this model found that a longer convalescence between ZIKV and DENV challenge was associated with higher and more durable antibody and T cell responses suggesting that ZIKV immune memory can contribute to protection against DENV (95).

## Dengue and ZIKA Vaccines in Development

**Figure 7:**
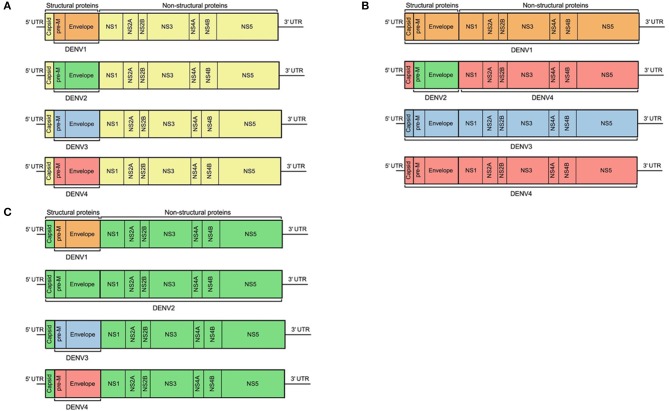
Design schematic of three dengue vaccines. **(A)** represents Sanofi's licensed vaccine, Dengvaxia®, **(B)** represents the NIH live attenuated tetravalent dengue vaccine candidate (TV003), and **(C)** represents Takeda's tetravalent dengue vaccine (TDV). Each color represents regions from different flaviviruses: yellow, YFV; orange, DENV1; green, DENV2; blue, DENV3; and red, DENV4.

There are currently three dengue vaccines that have either been tested in, or are currently in, phase III trials. At the time of writing the only licensed dengue vaccine is Sanofi-Pasteur's Dengvaxia® (CYD-TDV), a live-attenuated, chimeric, tetravalent vaccine, in which the genes encoding prM and E of the four DENV serotypes have been inserted into YFV-17D (Figure 7) (96). The vaccine was developed to produce DENV neutralizing antibodies in human subjects, but protection against disease is incomplete despite high levels of seroconversion (97, 98), and one trial found no efficacy at all against DENV2 (99). During longer follow up of clinical trial participants, it was observed that young children in the vaccinated group had excess hospital admissions due to dengue compared with the placebo group (100). Dengvaxia is most effective in individuals who are DENV-seropositive at the time of immunization, while in seronegative subjects the vaccine is not protective and increases the risk of severe disease (101). One hypothesis for failure to protect DENV-naïve subjects may be that the T cell response generated was directed against the yellow fever NS proteins present within the vaccine, rather than DENV NS proteins (68, 102). These observations suggest the need to determine the optimal T cell antigens and incorporate them into new vaccines. The other two dengue vaccines in development are based on full length DENV. One [Takeda, tetravalent dengue vaccine (TAK-003)] uses an attenuated strain of DENV2, with the prM and E genes of the other serotypes inserted into it (103). Regardless of dengue serostatus, TAK-003 elicited strong humoral responses against all four DENV serotypes (104), and generated a polyfunctional CD8+ T cell response to the non-structural proteins of DENV2, which cross-reacted against DENV1, DENV3, and DENV4 (105). Preliminary findings of a phase III trial show TAK-003 to have ~81% efficacy against symptomatic dengue (106), though protection against DENV3 was slightly lower. Therefore, a vaccine that induces better—and potentially more cross-reactive T cell responses also seems to have higher efficacy. However, these vaccines have not been compared directly and TAK-003 may still enhance disease in DENV naïve people, and could protect through a mechanism not involving CD8+ T cells. The other dengue vaccine in phase III trials is TV003, which is a tetravalent formulation of DENV1-3 with an additional chimeric DENV4 with the DENV2 prM and E genes inserted (Figure 7). Administration of TV003 induced a T cell response which predominantly targeted conserved epitopes of NS3 and NS5 (55) and was found to be immunogenic in subjects with prior flavivirus exposure (107). Field efficacy data are not yet available for TV003, but the vaccine protects against rash and viremia in a dengue human challenge model (108).

The majority of Zika vaccines are still in phase I/II trials (Table 2). These vaccines include DNA/mRNA, purified inactivated ZIKV, and recombinant virus-vectored vaccines; with most vaccine constructs containing the prM and E antigens as the main immunogen, as these proteins are targets for neutralizing antibodies (109). Three DNA-based vaccines (GLS-5700, VRC5283, and VRC5288) that have entered human testing show promising results, with one, VRC5283 advancing into phase II. VRC5283 and VRC5288 are both chimeric vaccines that utilize a JE virus (JEV) prM signal sequence followed by either the full-length E protein from wildtype ZIKV (VRC5283) or a modified E region in which the terminal 98 amino acids are exchanged with the analogous JEV sequence (VRC5288) (110). Both vaccines were shown to be immunogenic in mouse and NHP models (110) and a phase I trial found that vaccination with VRC5283 elicited neutralizing antibodies and cellular responses in all of the participants (111). A randomized placebo-controlled phase II study of VRC5283 is currently underway. A limitation of VRC5283 is whether the incorporation of a sequence from a different flavivirus (JEV prM) will provide protection against congenital Zika infection (111). To address this point, the vaccine was tested in a non-human primate pregnancy model. Vaccinated animals displayed fewer fetal losses and had reduced placental and fetal pathology; vaccine protection correlated with serum neutralizing antibody and antiviral T cell responses (112). However, these results may have to be considered cautiously as the model does not reflect early gestational exposure to ZIKV (112).

**Table 2:** Current Zika vaccines in clinical trial.

**Vaccine platform**	**Name**	**Immunogen**	**Adjuvant**	Dose^*^	**Sponsor**	**Phase I**	**Phase II**
DNA	VRC5283	prM-E	None	4 mg IM (Phase I) and 4 mg vs. 8 mg IM (Phase II)	NIAID/VRC	NCT02996461	NTC03110770
	VRC5288	prM-E	None	4 mg IM	NIAID/VRC	NCT02840487	–
	GLS5700	prM-E	None	1,2 mg ID	GeneOne Life Science Inovio Pharmaceuticals	NCT02809443 NCT02887482	–
mRNA	mRNA-1325	prM-E	None	–	Moderna Therapeutics	NCT03010489	–
Inactivated Virus	ZPIV	virion	Alum	5 μg IM	NIAID/WRAIR/BIDMC	NCT02963909 NCT02952833 NCT02937233	–
	BBV121	virion	Alum	2.5 μg vs. 5 μg vs. 10 μg IM	Bharat Biotech	CTRI/2017/05/008539	–
	PIZV	virion	Alum	2 μg vs. 5 μg vs. 10 μg IM	Takeda	NCT03343626	–
	VLA1601	virion	Alum	2.5 μg vs. 5 μg vs. 10 μg IM	Valneva Emergent Biosolutions	NCT03425149	–
Viral Vectored	MV-ZIKV	prM-E	None	Low dose vs. High dose IM	Themis Biosciences	NCT02996890	–
	Ad26.ZIKV.001	M-E	None	–	Janssen Vaccines	NCT03356561	–

* *IM, intramuscular; ID, intradermal*.

In addition to DNA-based vaccines, a ZIKV purified inactivated vaccine (ZPIV) based on the Puerto Rican strain PRVABC59 was found to be immunogenic at phase I with an acceptable safety profile (113). Vaccination with ZPIV elicited neutralizing antibody responses in the majority of tested individuals (113). One vaccine recipient from the trial produced cross-neutralizing antibodies to both ZIKV and DENV; responses which were linked to the individual's prior flavivirus exposure (114). These antibodies were shown to target the E domain I/III linker and could protect IFNAR^−/−^ mice challenged either with ZIKV or DENV-2 (114). The durability of these protective responses were only evaluated up to 8 weeks post-vaccination (114) and longer follow up is still needed to fully demonstrate the longevity of these responses.

Enhanced immunogenicity associated with viral vectors (115) make these attractive candidates for Zika antigen delivery. Of the vaccines in clinical testing, two use viral vectors: one an attenuated measles strain (116) and the other a replication-incompetent human adenovirus serotype 26 (Ad26) (117). A single immunization with the Ad26 construct containing the Zika prM and E (Ad26.ZIKV.M-Env) antigens was able to elicit protective humoral and cellular responses in mice and NHP (117). Ad26.ZIKV.M-Env also protected IFNAR^−/−^ dams and fetuses from ZIKV in a pregnancy model (118).

As discussed previously, all of vaccine constructs in clinical testing target structural proteins and may therefore not be optimal in their ability to induce cellular responses or boost pre-existing responses. Therefore, vaccines that include non-structural proteins should be considered, given that vaccination with non-structural proteins can induce cytotoxic T cell and polyfunctional helper T cell responses (119). Furthermore, given that non-structural proteins can elicit cross-reactive T cell responses, vaccines that incorporate these proteins may provide suitable priming for the development of memory responses during secondary flavivirus challenge (74, 94, 95, 109, 110, 115, 120–140).

## Conclusion

There still remains a need to develop Zika vaccines, and also to better understand the cross-reactivity of flavivirus immune responses so that this can be harnessed for the emergent flaviviruses of the future. Dengue infection appears to be protective against ZIKV through mechanisms mediated by cross-reactive T cell responses against NS proteins. Given that in general, non-structural proteins elicit the most cross-reactive responses, and that these responses are likely to be protective against other viruses beyond dengue, there is a strong argument for including non-structural proteins to act as CD8 T cell antigens in novel flavivirus vaccines. As the majority of the population in flavivirus endemic areas are repeatedly exposed, vaccines incorporating non-structural antigens may be more efficient and may require fewer doses due to the constant boosting of an existing T cell response. Finally, the degree of cross-reactivity seen with human CD8+ T cell responses to flaviviruses raises the possibility of engineering a single component containing T cell antigens that could be used in multiple vaccines, or even in a multivalent vaccine, provided suitable B cell antigens can be found.

## Author Contributions

KS contributed to the conceptualization of the article, wrote the first draft, and reviewed the manuscript. SL and LG contributed to conceptualization of the article and wrote the first draft of sections of the article. AG contributed viral homology measures and also to conceptualization of the article. DW and LT contributed to conceptualization of the article, contributed to early drafts, and reviewed the article. All authors reviewed the final draft.

### Conflict of Interest

The authors declare that the research was conducted in the absence of any commercial or financial relationships that could be construed as a potential conflict of interest.
